# Intolerance of uncertainty and psychological flexibility as predictors of mental health from adolescence to old age

**DOI:** 10.1007/s00127-024-02724-z

**Published:** 2024-07-09

**Authors:** Sakiko Okayama, Savannah Minihan, Jack L. Andrews, Sarah Daniels, Karina Grunewald, Matthew Richards, Weike Wang, Yasmin Hasan, Susanne Schweizer

**Affiliations:** 1 Department of Psychology, University of New South Wales, Kensington, Sydney, Australia; 2 University of Cambridge, Cambridge, UK

**Keywords:** Depression, Anxiety, Intolerance of uncertainty, Psychological flexibility, Adolescence

## Abstract

**Purpose:**

The COVID-19 pandemic brought with it significant social, economic and health uncertainties. These were proposed to impact young people more compared to adults, leading adolescents to report more mental health problems during the pandemic. The current study examined whether differences in cognitive risk (tolerance of uncertainty) and protective (psychological flexibility) factors accounted for age-related differences in depression and anxiety.

**Methods:**

These associations were investigated in the COVID-19 Risks Across the Lifespan (CORAL) cohort (*N* = 2280, 11–89 years).

**Results:**

The results showed that adolescents experienced greater intolerance of uncertainty and lower psychological flexibility compared to adults and older adults. Tolerance of uncertainty did not account for age-related differences in depression or anxiety. However, psychological flexibility conferred more protective advantage for anxiety in adults compared to adolescents.

**Conclusion:**

The observed age-related differences in risk and protective factors advance our understanding of developmental vulnerabilities to depression and anxiety. Implications for mental health interventions in the context of future pandemics are discussed.

**Supplementary Information:**

The online version contains supplementary material available at 10.1007/s00127-024-02724-z.

## Introduction

The COVID-19 pandemic had global health, economic and social impacts. Its stressors were accompanied with global increases in mental health problems [1], especially in adolescents (10–24 year-olds; [2]), who reported higher depression and anxiety symptoms compared to adults [3] and children [4]. In contrast, older adults reported lower depression and anxiety compared to younger adults, despite perceiving greater risk of death from a potential COVID-19 infection [5, 6]. Identifying risk factors (e.g., intolerance of uncertainty; IU) and protective factors (e.g., psychological flexibility; PF) that contribute to these age-related differences in the mental health impacts of the pandemic is critical to inform mental health interventions for potential future disease outbreaks.

## Intolerance of uncertainty and mental health across the lifespan

A global pandemic raises uncertainties across domains including health, such as virus severity, long-term health impacts and vaccine availability and efficacy, along with on-going economic and social uncertainty. Individuals vary in their capacity to tolerate uncertainty. High IU, the tendency to react negatively at a cognitive, affective and behavioural level in response to uncertainty, is a transdiagnostic risk factor for mental health problems [7]. During the pandemic, high IU was associated with increased mental health problems in children and adolescents [4], adults [8, 9], and older adults [10, 11]. However, it is unclear whether IU differentially impacts mental health across the lifespan.

IU changes across development. The decrease in IU from adulthood to older age [12] is supported by the dual-process framework of aging, which suggests that older adults tolerate uncontrollability well because they are used to adapting to functional losses, such as cognitive and health decline [13]. Thus, low IU in older adults may partially account for their relatively good mental health during COVID-19. Like older adults, adolescents show less behavioural avoidance of uncertainty compared to adults [14, 15] and greater readiness to explore uncertain environments [16]. This greater exploration of, and exposure to, uncertain environments is proposed to confer benefit later in development [17]. However, this appears in direct contrast with the greater mental health impact which high uncertainty during the pandemic had on adolescent mental health [18]. A possible account for this apparent paradox is that, despite a developmental readiness to approach uncertainty, the cognitive and affective costs of engaging uncertainty on adolescents may be higher than in other age groups [19]. However, age differences in cognitive and affective responses to uncertainty and how these relate to mental health remain largely unexplored.

## Psychological flexibility and mental health across the lifespan

Individuals’ perceived need for control and avoidance of uncertainty may reinforce rigid behaviours and cognitions (e.g., repeatedly checking for news about the virus) during periods of great uncertainty such as the pandemic [20]. However, mental health resilience during times of adversity has been shown to depend on PF instead [21]. PF as defined here, refers to individuals’ ability to accept and adopt their cognitive, affective and behavioural responses to environmental changes (for a review of different definitions of PF see Cherry et al. [22]).

PF has been associated with fewer symptoms of depression and anxiety in adults [23] and older adults [24]. During COVID-19, PF had a protective effect on mental health in adults [e.g., [25], and preliminary evidence suggests that PF may similarly confer a protective advantage on adolescents’ mental health [26]. Despite its robust associations with mental health, less is known about the development of PF.

Interestingly, older adults show better PF compared to younger adults [24], despite overall lower cognitive flexibility in older age [27]. In adolescence, there is protracted development of the neural substrates of cognitive flexibility [28], which matures in tandem with its underlying cognitive correlates [29]. This may in turn partially account for increased mental health vulnerability in adolescents, especially during times of prolonged stress such as the COVID-19 pandemic.

PF may also interact with IU to impact mental health. Specifically, PF may serve as a protective factor for mental health in individuals who are high on IU but are able to flexibly pursue meaningful goals and values rather than becoming preoccupied with uncertainty. Indeed, studies have found that PF moderated the associations between IU and mental health during the pandemic [e.g., [30], although these interactions have mostly been investigated cross-sectionally. Together, the literature points to IU and PF as risk and protective factors for mental health that may differentially influence individuals across the lifespan.

## The current study

The current study aimed to examine the role of IU and PF in the association between age and mental health during COVID-19. Accordingly, data from the COVID-19 Risks Across the Lifespan (CORAL) cohort, which measured participants three times between May 2020 and April 2021, was used. Previous work in this cohort showed that younger, relative to older, participants reported greater mental health problems [3] and greater negative affect [31] at all three timepoints (T1-T3). In the current study we investigated whether these age-related differences were associated with IU and/or PF. We tested the pre-registered hypotheses that: There would be age-related differences in levels of cognitive risk (IU; *H1a*) and protective (PF; *H1b*) factors for mental health, with IU showing a quadratic and PF a linear association with age at T1. Second, the association between age group and mental health problems would be partially accounted for by IU (*H2a*) and PF (*H2b*), cross-sectionally at T1 and across time from T1 to T3. Finally, IU and PF were predicted to interact, with change in PF from T1 to T3 mediating the association between IU at T1 and mental health problems at T3.

## Methods

### Participants

The present analyses included 2,280 participants from the CORAL cohort (*N* = 3,208), with a mean age of 38.98 (*SD* = 16.99) years, 89.82% of whom identified as female and 69.39% as high socioeconomic status (Table S1). Participants were based in the US, UK and Australia. Of the total participant pool, 84.52% identified as White, 4.34% as Asian, 3.07% as mixed, 1.75% as Hispanic, 0.7% as Black, 0.39% as Aboriginal or Torres Strait Islander and 3.95% with another ethnic background. For a full breakdown of participant ethnicities, details on recruitment and eligibility criteria see supplementary materials (SM). Table S2 includes descriptives and correlations between study variables. For attrition analyses as a function of demographic characteristics, see Minihan et al. [3].

## Measures

### Intolerance of uncertainty

IU was measured at T1 using the Intolerance of Uncertainty Scale – Short Form [IUS-12; 30]. Participants rated their agreement with 12 items (e.g., “unforeseen events upset me greatly”) in relation to the past week on a 5-point scale (1 = *not at all*, 5 = *very much*). The scale showed good internal consistency in the present study, *ωT* = 0.94.

### Psychological flexibility

PF was measured at all timepoints using the Mental Flexibility Questionnaire–State [MFQ; 31]. Participants rated their agreement with eight items (e.g., “I have been good at accepting change”) in relation to the past week on a 6-point scale (1 = *strongly disagree,* 6 = *strongly agree)*. The scale had good internal consistency in the present study, *ωT* = 0.92-0.93.

### Depression

Depressive symptoms were measured at all timepoints using the eight-item Patient Health Questionnaire [PHQ-8; [34]. Participants rated how often they were bothered by such things as “little interest or pleasure in doing things” across the past two weeks, on a 4-point scale (0 = *not at all*, 3 = *nearly every day).* The PHQ-8 had good internal consistency in the present study, *ωT* = 0.93-0.94.

### Anxiety

Symptoms of anxiety were assessed at all timepoints with the seven-item Generalized Anxiety Disorder Scale [GAD-7; [35]. Participants rated how often they were bothered by such things as “feeling nervous, anxious or on edge” during the past two weeks, on a 4-point scale (0 = *not at all,* 3 = *nearly every day)*. The GAD-7 showed good internal consistency in the present study, *ωT* 0.95-0.96).

### COVID-19 risk

At all timepoints, participants provided binary responses to indicate whether they or a member of their household had been quarantined due to suspected or confirmed COVID-19 infection; whether they had been hospitalized because of COVID-19; and whether they personally knew someone who had been diagnosed with, hospitalized because of, or died from COVID-19. A weighted composite score was computed (see SM) and included in all analyses as a covariate, to control for individual differences in COVID-19 exposure and health risk.

## Procedure

The CORAL study was administered online on the Qualtrics platform at three timepoints: T1 (May 5, 2020–September 30, 2020), T2 (August 5, 2020–January 29, 2021), and T3 (November 5, 2020–April 9, 2021). Participants who answered at least 65% of the T1 survey were invited to participate in T2 and T3. All participants provided informed consent prior to participating and participants under 18 years additionally required consent from a parent/guardian.

## Data analysis

Two general linear models were specified to investigate the effect of age group on IU (H1a) and PF (H1b). To investigate the predicted effect of IU (H2a) and PF (H2b) on the association between age and mental health, separate models were specified for depression and anxiety as outcomes. For the cross-sectional analyses at T1, general linear models were specified with age group, IU/PF as predictors. For the longitudinal analyses, mixed-effects models were specified, with time included as a fixed effect, age group and IU as time-invariant fixed effects, PF as a time-variant fixed effect, and participant ID as a random effect. Interactions between age group and IU/PF were also included. To test whether PF would account for the association between IU and mental health problems, mediation models were specified. IU was included as the predictor and change in PF (T3 – T1) as the mediator. In a first model, T3 depression was included as the outcome and T1 depression as a covariate. In a second model, T3 anxiety was included as the outcome and T1 anxiety as a covariate.

Analyses were conducted in R (Version 4.1.3), following a preregistered analysis plan (osf.io/4xd76). For specific packages used, see SM. For analyses, age group was coded as a numeric variable (1 = early-to-mid adolescents (11–17 years); 2 = late adolescents (18–24 years); 3 = adults (25–64 years); 4 = older adults (65 + years)). Although group sizes were uneven across the age bins (SM Table 1), mixed-effects models are able to handle uneven group sizes [36]. For analyses with depression or anxiety as the outcome, inferences were based on one-tailed *p*-values with Bonferroni-corrected significance threshold set at *p* < 0.025 (*α*/2) to correct for examining two mental health outcomes. For all other preregistered analyses, inferences were based on one-tailed *p*-values with significant threshold set at *p* < 0.05.

## Results

### Hypothesis 1: age-related differences in intolerance of uncertainty and psychological flexibility

**Fig. 1:**
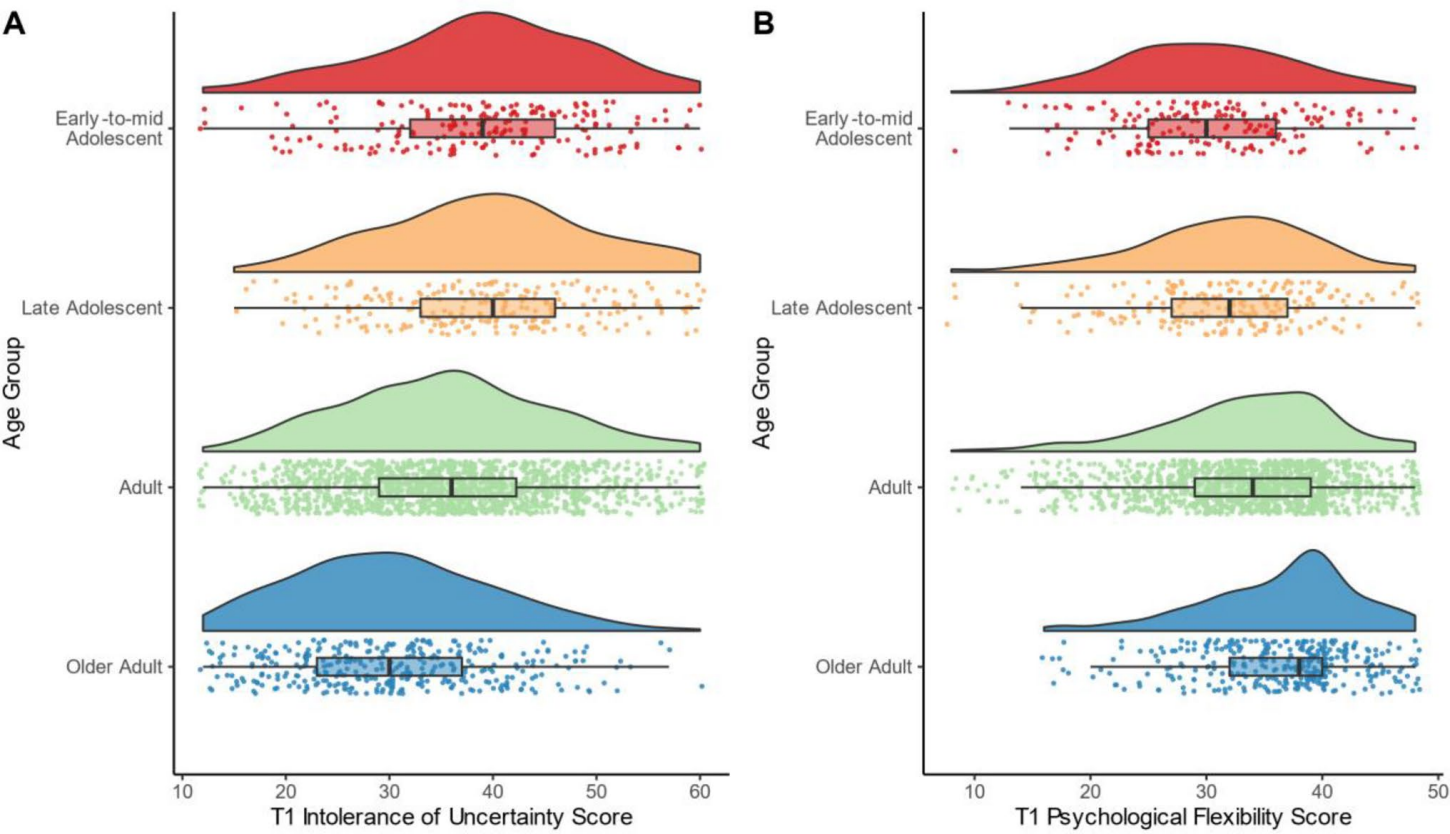
Age-Related Differences in Intolerance of Uncertainty and Psychological Flexibility at T1. Note. T1 occurred between May 5, 2020 and September 30, 2020. The following age groups were used: early-to-mid adolescent (11–17 years), late adolescent (18–24 years), adult (25–64 years), and older adult (65 + years). Boxplots show the median and interquartile range. Panel **A**: Age differences in intolerance of uncertainty, measured with the 12-item Intolerance of Uncertainty Scale–Short Form [32]. Panel **B**: Age differences in psychological flexibility, measured with the 8-item Mental Flexibility Questionnaire–State [33]

Age-related differences in IU and PF were observed at T1. For IU, a model that included linear and quadratic terms for age group provided a better fit compared to a linear-only model (*F*(1, 2276) = 28.52, *p* < 0.001). The significant linear effect of age group on IU (H1a; *F*(3, 2276) = 52.82, *p* < 0.001, *R*^*2*^ = 0.06) showed decreasing IU across age groups (SM Table 3). There was also a significant quadratic effect of age group on IU (SM Table 3; Fig. 1A), with IU peaking in late adolescence and then decreasing into older age. Furthermore, there was a significant linear effect of age group on PF, (H1b; *F*(2, 2123) = 48.43, *p* < 0.001, *R*^*2*^ = 0.04), with PF increasing as a function of age group (SM Table 3; Fig. 1B).

### Hypothesis 2: the role of intolerance of uncertainty and psychological flexibility in the association between age and mental health problems

In contrast to H2a, age group did not interact significantly with IU to predict symptoms of depression or anxiety, neither cross-sectionally at T1 (SM Table 4) nor longitudinally from T1 to T3 (SM Table 5). Similarly, in contrast to H2b, age group and PF did not significantly interact to predict symptoms of depression at T1 (SM Table 4) nor from T1 to T3 (SM Table 5). However, age group did significantly interact with PF to predict anxiety symptoms at T1 (SM Table 4) and from T1 to T3 (SM Table 5).

**Fig. 2:**
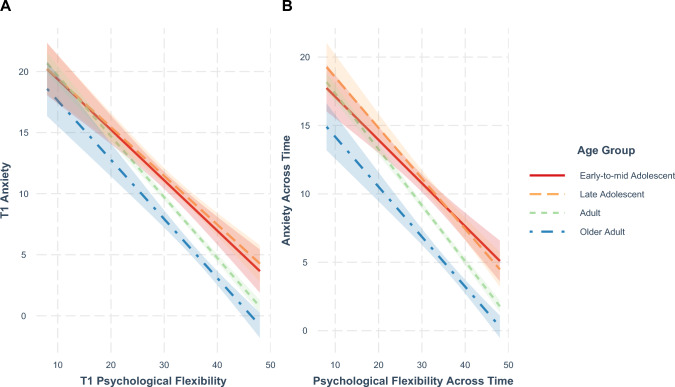
The Association between Psychological Flexibility and Anxiety as a Function of Age Group. Note. T1 occurred between May 5, 2020 and September 30, 2020, T2 occurred between August 5, 2020 and January 29, 2021 and T3 occurred between November 5, 2020 and April 9, 2021. Psychological flexibility was measured with the 8-item mental flexibility questionnaire–state [33]. Anxiety was measured with the 7-item Generalized Anxiety Disorder Scale [35]. The following age groups were used: early-to-mid adolescent (11–17 years), late adolescent (18–24 years), adult (25–64 years), and older adult (65 + years). Panel **A**: Cross-sectional association between psychological flexibility and symptoms of anxiety at T1. Panel **B**: Longitudinal association between psychological flexibility and symptoms of anxiety across time (T1 to T3)

The significant interaction between age group and PF was further examined by investigating the effect of PF on anxiety in each age group separately, cross-sectionally at T1 and across time from T1 to T3. At T1 (Fig. 2A) the protective effect of PF on anxiety was stronger in adults (*b* = − 0.50, *SE* = 0.02, 97.5% CI [− 0.54, − 0.46], *p* < 0.001) and older adults (*b* = − 0.49, *SE* = 0.03, CI [− 0.56, − 0.41], *p* < 0.001) compared to early-to-mid adolescents (*b* = − 0.41, *SE* = 0.05, CI [− 0.52, − 0.30], *p* < 0.001) and late adolescents (*b* = − 0.40, *SE* = 0.05, CI [− 0.51, − 0.29], *p* < 0.001). The protective effect of T1–T3 PF on T1–T3 anxiety (Fig. 2B) was again strongest in adults (*b* = − 0.41, *SE* = 0.01, 97.5% CI [− 0.44, − 0.38], *p* < 0.001) compared to other age groups (early-to-mid adolescents: *b* = − 0.34, *SE* = 0.04, CI [− 0.44, − 0.24], *p* < 0.001; late adolescents: *b* = − 0.35, *SE* = 0.04, CI [− 0.44, − 0.26], *p* < 0.001; older adults: *b* = − 0.36, *SE* = 0.02, CI [− 0.42, − 0.30], *p* < 0.001).

### Hypothesis 3: the mediating role of pf in the association between intolerance of uncertainty and mental health

To investigate whether change in PF across time mediated the association between IU and mental health problems, mediation models were specified. The longitudinal model included T1 IU as predictor, change in PF across time (T3–T1) as mediator, T3 depression as outcome, and T1 depression as covariate. The analysis was repeated for anxiety. Change in PF from T1 to T3 did not significantly mediate the association between T1 IU and T3 depression (indirect effect: b* = 0.000, *SE* = 0.001, *z* = 0.06, *p* = 0.954) or T3 anxiety (indirect effect: b* = − 0.000, *SE* = 0.001, *z* = − 0.100, *p* = 0.920).

## Discussion

The pandemic has disproportionally affected adolescent mental health, exacerbating existing trends of increasing mental health problems observed over recent decades. In the present study, we examined whether age-related differences in the way in which individuals tolerate uncertainty—a key characteristic of the COVID-19 pandemic—and individuals’ ability to flexibly adapt their thoughts or behaviours in the context of adversity, may account for age-related differences in mental health problems across a year of the pandemic.

### Age-related differences in intolerance of uncertainty and psychological flexibility

In contrast with our predictions, IU was greater in adolescents compared to adults, with IU peaking in late adolescence. The existing literature which has shown less uncertainty avoidance in adolescence compared to adulthood has studied behavioural responses to uncertainty [12, 13, e.g., 15]. Indeed, adolescents (11–17 years) reported higher IU compared to the adult normative scores using self-report measures. The uncertainty reflected in behavioural tasks may be less threatening (e.g., small monetary gains and losses) than real life uncertainties where the stakes are higher (e.g., social, health, and economic uncertainty). Indeed, task-based and self-report measures of IU show only small to moderate correlations in adults [37]. Alternatively, the discrepancy observed is a true reflection of conflicting goal states in adolescence. That is, while it is developmentally beneficial to explore the adult environment [17], our results suggest this may come at the cost of psychological distress due to high cognitive IU in adolescents. If replicated, this conflict may partially account for high levels of negative affect observed in adolescents compared to adults, especially during times of high uncertainty [31].

Further, adolescence is a period of both heightened threat sensitivity and heightened reward sensitivity, which may contribute to the observed age-related differences in response to uncertainty [19]. Specifically, threat sensitivity and reward sensitivity may be differentially activated in the face of uncertainty in adolescence, such that when uncertainty signals high potential gain (e.g., peer acceptance), adolescents tend to show high tolerance of uncertainty (e.g., increased risk-taking behaviours; [38]). In contrast, when uncertainty signals high potential threat/loss (e.g., social isolation), threat sensitivity may override reward sensitivity and lead to higher intolerance of uncertainty. This may be especially true for late adolescents (18–24 years), who typically experience important transitions in educational, employment, and/or housing contexts, which were likely disrupted by COVID-19.

Supporting our hypothesis, older adults reported the lowest IU. This is consistent with the socioemotional theory, which posits that people become less concerned with future-oriented goals such as reducing uncertainty as they age [39].

The observed increase in PF with age is consistent with the selection, optimisation, and compensation theory, which suggests that older adults compensate for diminished cognitive resources by flexibly pursuing new goals or relying more on previously under-used resources such as social support [40]. The lower PF in younger adolescents potentially reflects the ongoing development of its proposed cognitive substrate, affective control, which continues to develop into late adolescence [28].

### Intolerance of uncertainty and psychological flexibility impact mental health across age

We found that IU did not account for age-related differences in mental health. That is, the relationship between IU and mental health outcomes was the same for younger and older individuals. One reason for this lack of age-related differences may be that age-related variance in mental health problems might be tied to more specific domains of uncertainty (e.g., health, social, economic). For example, it has been proposed that adolescents may be especially impacted by social uncertainties [18], which may have been particularly salient in the context of the pandemic (e.g., when they will next see their friends).

In contrast to IU, PF did partially account for the association between age and anxiety, such that this association was stronger in adults compared to other age groups. If adolescents have low PF, and PF is also less protective against anxiety during adolescence compared to adulthood, this may partially explain high rates of adolescent anxiety during the pandemic [31]. The greater protective effect of PF in adults compared to older adults may be that other mechanisms such as social support are more predictive of mental wellbeing in older adults. Together, these findings suggest that improving PF and reducing IU, especially in young people, may benefit population mental health, especially during future pandemics, or periods of significant uncertainty.

## Limitations

These results should be interpreted within the context of several limitations. First, the sample was a non-representative convenience sample. The majority of participants were female, limiting the generalisability of the findings to other genders. A further limitation is that only health-related COVID-19 stressors were controlled for. Future work should seek to examine whether distinct types of uncertainty (e.g., social, financial, health) differentially impact mental health outcomes across the lifespan.

## Conclusion

Adolescents appear to experience greater cognitive IU and lower PF compared to adults and older adults. We found no evidence for the argument that age-related differences in individuals’ ability to tolerate uncertainty accounted for more symptoms of depression and anxiety in young people compared to adults. However, the protective effect of PF varied across age during the COVID-19 pandemic, with PF conferring the greatest benefit to adults. Interventions aimed at increasing PF and reducing IU may improve mental health across all age groups during future pandemics.

## Supplementary Information

Below is the link to the electronic supplementary material.

Supplementary file1 (DOCX 68 KB)

## Data Availability

Data availability on request. We have ethical approval to share data with researchers who request the data and confirm they will be using it in accordance with the Helsinki Declaration of Ethical Principles for Research.

## References

[1] Witteveen AB, Young SY, Cuijpers P et al (2023) COVID-19 and common mental health symptoms in the early phase of the pandemic: an umbrella review of the evidence. Plos Med 20:e1004206. 10.1371/journal.pmed.1004206 37098048 10.1371/journal.pmed.1004206 PMC10129001

[2] Sawyer SM, Azzopardi PS, Wickremarathne D, Patton GC (2018) The age of adolescence. Lancet child Adolesc Health 2(3):223–228 30169257 10.1016/S2352-4642(18)30022-1

[3] Minihan S, Orben A, Songco A et al (2022) Social determinants of mental health during a year of the COVID-19 pandemic. Dev Psychopathol. 10.1017/S0954579422000396 35796203 10.1017/S0954579422000396 PMC7615306

[4] Raymond C, Provencher J, Bilodeau-Houle A et al (2022) A longitudinal investigation of psychological distress in children during COVID-19: the role of socio-emotional vulnerability. Eur J Psychotraumatology 13:2021048. 10.1080/20008198.2021.2021048 10.1080/20008198.2021.2021048 PMC8788367 35087645

[5] Bruine De Bruin W (2021) Age differences in COVID-19 Risk perceptions and mental health: evidence from a national U.S. survey conducted in march 2020. J Gerontol Ser B 76:e24–e29. 10.1093/geronb/gbaa074 10.1093/geronb/gbaa074 PMC7542924 32470120

[6] Pieh C, Budimir S, Probst T (2020) The effect of age, gender, income, work, and physical activity on mental health during coronavirus disease (COVID-19) lockdown in Austria. J Psychosom Res 136:110186. 10.1016/j.jpsychores.2020.110186 32682159 10.1016/j.jpsychores.2020.110186 PMC7832650

[7] Osmanağaoğlu N, Creswell C, Dodd HF (2018) Intolerance of uncertainty, anxiety, and worry in children and adolescents: a meta-analysis. J Affect Disord 225:80–90. 10.1016/j.jad.2017.07.035 28802117 10.1016/j.jad.2017.07.035

[8] Andrews JL, Li M, Minihan S et al (2023) The effect of intolerance of uncertainty on anxiety and depression, and their symptom networks, during the COVID-19 pandemic. BMC Psychiatry 23:261. 10.1186/s12888-023-04734-8 37069541 10.1186/s12888-023-04734-8 PMC10109227

[9] Rettie H, Daniels J (2021) Coping and tolerance of uncertainty: Predictors and mediators of mental health during the COVID-19 pandemic. Am Psychol 76:427–437. 10.1037/amp0000710 32744841 10.1037/amp0000710

[10] Bavolar J, Kacmar P, Hricova M et al (2023) Intolerance of uncertainty and reactions to the COVID-19 pandemic. J Gen Psychol 150:143–170. 10.1080/00221309.2021.1922346 34006200 10.1080/00221309.2021.1922346

[11] Köverová M, Ráczová B, Kováčová Holevová B (2021) Predictors of anxiety, stress, and concern of COVID-19 infection in older adults during the first and the second waves of the COVID-19 pandemic in Slovakia. Gerontol Geriatr Med 7:233372142110476. 10.1177/23337214211047642 10.1177/23337214211047642 PMC8511907 34660848

[12] Basevitz P, Pushkar D, Chaikelson J et al (2008) Age-Related Differences in worry and related processes. Int J Aging Hum Dev 66:283–305. 10.2190/AG.66.4.b 18507331 10.2190/AG.66.4.b

[13] Brandtstädter J (2009) Goal pursuit and goaladjustment: Self-regulation and intentional self-development in changing developmental contexts. Adv Life CourseRes 14(1–2):52–62

[14] Nussenbaum K, Martin RE, Maulhardt S, et al (2022) Novelty and uncertainty differentially drive exploration across development 10.7554/eLife.84260 PMC10431916 37585251

[15] van den Bos W, Hertwig R (2017) Adolescents display distinctive tolerance to ambiguity and to uncertainty during risky decision making. Sci Rep 7:40962. 10.1038/srep40962 28098227 10.1038/srep40962 PMC5241878

[16] Lloyd A, McKay R, Sebastian CL, Balsters JH (2021) Are adolescents more optimal decision-makers in novel environments? Examining the benefits of heightened exploration in a patch foraging paradigm. Dev Sci 24:e13075. 10.1111/desc.13075 33305510 10.1111/desc.13075

[17] Ciranka S, van den Bos W (2021) Adolescent risk-taking in the context of exploration and social influence. Dev Rev 61:100979. 10.1016/j.dr.2021.100979

[18] Schweizer S, Lawson RP, Blakemore S-J (2023) Uncertainty as a key driver of the youth mental healt crisis. Curr Opin Psychol. 10.1016/j.copsyc.2023.101657 37517166 10.1016/j.copsyc.2023.101657

[19] Baker AE, Galván A (2020) Threat or thrill? The neural mechanisms underlying the development of anxiety and risk taking in adolescence. Dev Cogn Neurosci 45:100841. 10.1016/j.dcn.2020.100841 32829216 10.1016/j.dcn.2020.100841 PMC7451699

[20] Einstein DA (2014) Extension of the transdiagnostic model to focus on intolerance of uncertainty: a review of the literature and implications for treatment. Clin Psychol 21:280–300. 10.1111/cpsp.12077 10.1111/cpsp.12077 PMC4204511 25400336

[21] Parsons S, Kruijt A-W, Fox E (2016) A cognitive model of psychological resilience. J Exp Psychopathol 7:296–310. 10.5127/jep.053415

[22] Cherry KM, Hoeven EV, Patterson TS, Lumley MN (2021) Defining and measuring psychological flexibility: a narrative scoping review of diverse flexibility and rigidity constructs and perspectives. Clin Psychol Rev 84:101973. 10.1016/j.cpr.2021.101973 33550157 10.1016/j.cpr.2021.101973

[23] Kashdan TB, Disabato DJ, Goodman FR et al (2020) Understanding psychological flexibility: a multimethod exploration of pursuing valued goals despite the presence of distress. Psychol Assess 32:829–850. 10.1037/pas0000834 32614192 10.1037/pas0000834

[24] Plys E, Jacobs ML, Allen RS, Arch JJ (2023) Psychological flexibility in older adulthood: a scoping review. Aging Ment Health 27:453–465. 10.1080/13607863.2022.2036948 35168415 10.1080/13607863.2022.2036948 PMC9376200

[25] McCracken LM, Badinlou F, Buhrman M, Brocki KC (2021) The role of psychological flexibility in the context of COVID-19: associations with depression, anxiety, and insomnia. J Context Behav Sci 19:28–35. 10.1016/j.jcbs.2020.11.003

[26] Puolakanaho A, Muotka JS, Lappalainen R et al (2023) Adolescents’ stress and depressive symptoms and their associations with psychological flexibility before educational transition. J Adolesc 95:990–1004. 10.1002/jad.12169 36960576 10.1002/jad.12169

[27] Kupis L, Goodman ZT, Kornfeld S et al (2021) Brain dynamics underlying cognitive flexibility across the lifespan. Cereb Cortex 31:5263–5274. 10.1093/cercor/bhab156 34145442 10.1093/cercor/bhab156 PMC8491685

[28] Dajani DR, Uddin LQ (2015) Demystifying cognitive flexibility: Implications for clinical and developmental neuroscience. Trends Neurosci 38:571–578. 10.1016/j.tins.2015.07.003 26343956 10.1016/j.tins.2015.07.003 PMC5414037

[29] Szemenyei E, Reinhardt M, Szabó E et al (2020) Measuring psychological inflexibility in children and adolescents: evaluating the avoidance and fusion questionnaire for youth. Assessment 27:1810–1820. 10.1177/1073191118796558 30198319 10.1177/1073191118796558

[30] Inozu M, Gök BG, Tuzun D, Haciomeroglu AB (2022) Does cognitive flexibility change the nature of the relationship between intolerance of uncertainty and psychological symptoms during the COVID-19 outbreak in Turkey? Curr Psychol. 10.1007/s12144-021-02450-8 35002185 10.1007/s12144-021-02450-8 PMC8723801

[31] Minihan S, Songco A, Fox E et al (2023) Affect and mental health across the lifespan during a year of the COVID-19 pandemic: the role of emotion regulation strategies and mental flexibility. Emotion. 10.1037/emo0001238 37199936 10.1037/emo0001238 PMC11064816

[32] Carleton RN, Norton MAPJ, Asmundson GJG (2007) Fearing the unknown: a short version of the intolerance of uncertainty scale. J Anxiety Disord 21:105–117. 10.1016/j.janxdis.2006.03.014 16647833 10.1016/j.janxdis.2006.03.014

[33] Parsons S, Todorovic A, Lim MC et al (2022) Data and protocol for the oxford achieving resilience during covid-19 (ARC) study. J Open Psychol Data 10:4. 10.5334/jopd.56

[34] Kroenke K, Strine TW, Spitzer RL et al (2009) The PHQ-8 as a measure of current depression in the general population. J Affect Disord 114:163–173. 10.1016/j.jad.2008.06.026 18752852 10.1016/j.jad.2008.06.026

[35] Spitzer RL, Kroenke K, Williams JBW, Löwe B (2006) A brief measure for assessing generalized anxiety disorder: the GAD-7. Arch Intern Med 166:1092. 10.1001/archinte.166.10.1092 16717171 10.1001/archinte.166.10.1092

[36] Pinheiro JC (2014) Linear Mixed Effects Models for Longitudinal Data. In: Pinheiro JC (ed) Wiley StatsRef: Statistics Reference Online. Wiley

[37] Carleton RN, Duranceau S, Shulman EP, Zerff M, Gonzales J, Mishra S (2016) Self-reported intolerance of uncertainty and behavioural decisions. J Behav Ther Exp Psychiatry 51:58–65 26788617 10.1016/j.jbtep.2015.12.004

[38] Chein J, Albert D, O’Brien L, Uckert K, Steinberg L (2011) Peers increase adolescent risk taking by enhancing activity in the brain’sreward circuitry. Dev Sci 2:F1–10. 10.1111/j.1467-7687.2010.01035.x 10.1111/j.1467-7687.2010.01035.x PMC3075496 21499511

[39] Carstensen LL (2006) The influence of a sense of time on human development. Science 312(5782):1913–1915 16809530 10.1126/science.1127488 PMC2790864

[40] Freund AM (2008) Successfulaging as management of resources: The role of selection, optimization, and compensation. Res Human Dev 5(2):94–106

