# Supplementary material for: Intolerance of uncertainty and psychological flexibility as predictors of mental health from adolescence to old age
Source: Soc Psychiatry Psychiatr Epidemiol. 2024 Jul 9;59(12):2361–8. doi: 10.1007/s00127-024-02724-z (PMC11522106; doi:10.1007/s00127-024-02724-z)
Supplement: Supplementary file 1 — Supplementary file1 (DOCX 68 KB) [file 127_2024_2724_MOESM1_ESM.docx]

**Intolerance of Uncertainty and Psychological Flexibility as Predictors of Mental Health from Adolescence to Old Age**

Sakiko Okayama^1^, Savannah Minihan^1^, Jack L. Andrews^1^, Sarah Daniels^1^, Karina Grunewald^1^, Matthew Richards^1^, Weike Wang^1^, Yasmin Hasan^1^, CORAL, & Susanne Schweizer^1,2^

^1^University of New South Wales, ^2^University of Cambridge

**Corresponding Author**

A/Prof Susanne Schweizer

University of New South Wales, Department of Psychology

Kensington, Sydney, Australia

Email: [s.schweizer@unsw.edu.au](mailto:s.schweizer@unsw.edu.au)

**Supplementary Materials**

**Supplementary Method**

**Participants**

A total of 3,208 participants consented to and were eligible to participate in the wider CORAL study. For the present study, participants were excluded from analyses if they had additional duplicate data (T1: *n* = 9, T2: *n* = 21, T3: *n* = 13), failed two or more attention check items (T1: *n* = 8, T3: *n* = 1), did not provide data on the Intolerance of Uncertainty Scale at T1 (*n* = 904), or did not provide their age at T1 (*n* = 24). Of the 2,280 participants included in the present analyses, 890 commenced T2 and 822 commenced T3.

Participants were recruited online through social media advertisements (e.g., Facebook), conventional media, online research platforms (e.g., MQ Participate, COVID Minds), online forums, and through relevant organisations (e.g., schools, mental health organisations, parenting organisations). Participants were eligible to participate in CORAL if they had capacity to provide informed consent; were at least 11 years old; resided in Australia, the UK, or the USA; were fluent in English; and had no current or past neurological disorder, neurodevelopmental disorder, or traumatic brain injury. At T1, T2, and T3, one in every 100 participants received an AUD $100 (£50/ USD $60) gift card. Additionally, each participant who completed 100% of the T2 and T3 surveys received an AUD $10 (£5/ USD $6) gift card.

**SM Table 1**

*Participant Demographic Characteristics*

| Participant demographic characteristics | *n* | % |
| --- | --- | --- |
| Age group |  |  |
| Early-to-mid adolescents (11–17 years) | 193 | 8.46 |
| Late adolescents (18–24 years) | 228 | 10 |
| Adults (25–64 years) | 1512 | 66.32 |
| Older adults (65–100 years) | 347 | 15.22 |
| Gender |  |  |
| Female | 2048 | 89.82 |
| Male | 206 | 9.04 |
| Other | 20 | 0.88 |
| Prefer not to say | 6 | 0.26 |
| Country |  |  |
| United Kingdom | 1029 | 45.13 |
| United States of America | 679 | 29.78 |
| Australia | 572 | 25.09 |
| Ethnicity |  |  |
| White | 1927 | 84.52 |
| Asian | 99 | 4.34 |
| Other | 90 | 3.95 |
| Mixed | 70 | 3.07 |
| Hispanic | 40 | 1.75 |
| Black | 16 | 0.7 |
| Aboriginal or Torres Strait Islander | 9 | 0.39 |
| Prefer not to say | 28 | 1.23 |
| Missing | 1 | 0.04 |
| Socioeconomic status^a^ |  |  |
| High | 1582 | 69.39 |
| Average | 623 | 27.32 |
| Low | 4 | 0.18 |
| Missing | 71 | 3.11 |
| History of mental health diagnosis |  |  |
| Yes | 828 | 36.32 |
| No | 1452 | 63.68 |

^a^ Socioeconomic status (SES) was indexed using participants’ highest educational qualification, such that university = high SES, high school or professional/vocational training = average SES, and primary school = low SES. For participants under 18 years, the average of their parents’ highest educational qualification was used.

**SM Table 2**

*Descriptive Characteristics and Bivariate Correlations Between Study Variables*

| Variable | *M* | *SD* | 1 | 2 | 3 | 4 | 5 | 6 | 7 | 8 | 9 | 10 |
| --- | --- | --- | --- | --- | --- | --- | --- | --- | --- | --- | --- | --- |
|  |  |  |  |  |  |  |  |  |  |  |  |  |
| 1. Age | 38.98 | 16.99 |  |  |  |  |  |  |  |  |  |  |
|  |  |  |  |  |  |  |  |  |  |  |  |  |
| 2. T1 Intolerance of Uncertainty | 35.45 | 10.35 | –.25** |  |  |  |  |  |  |  |  |  |
|  |  |  | [–.29, –.21] |  |  |  |  |  |  |  |  |  |
|  |  |  |  |  |  |  |  |  |  |  |  |  |
| 3. T1 Psychological Flexibility | 33.46 | 7.52 | .19** | –.56** |  |  |  |  |  |  |  |  |
|  |  |  | [.15, .23] | [–.59, –.53] |  |  |  |  |  |  |  |  |
|  |  |  |  |  |  |  |  |  |  |  |  |  |
| 4. T2 Psychological Flexibility | 33.91 | 7.46 | .19** | –.43** | .65** |  |  |  |  |  |  |  |
|  |  |  | [.12, .26] | [–.48, –.37] | [.61, .69] |  |  |  |  |  |  |  |
|  |  |  |  |  |  |  |  |  |  |  |  |  |
| 5. T3 Psychological Flexibility | 33.26 | 7.65 | .23** | –.47** | .63** | .62** |  |  |  |  |  |  |
|  |  |  | [.16, .30] | [–.52, –.41] | [.58, .67] | [.56, .68] |  |  |  |  |  |  |
|  |  |  |  |  |  |  |  |  |  |  |  |  |
| 6. T1 Depressive symptoms | 9.33 | 6.32 | –.26** | .54** | –.60** | –.50** | –.47** |  |  |  |  |  |
|  |  |  | [–.30, –.22] | [.51, .56] | [–.63, –.57] | [–.55, –.44] | [–.53, –.41] |  |  |  |  |  |
|  |  |  |  |  |  |  |  |  |  |  |  |  |
| 7. T2 Depressive symptoms | 8.04 | 6.26 | –.25** | .45** | –.48** | –.58** | –.49** | .74** |  |  |  |  |
|  |  |  | [–.32, –.19] | [.39, .50] | [–.53, –.43] | [–.63, –.53] | [–.56, –.41] | [.71, .77] |  |  |  |  |
|  |  |  |  |  |  |  |  |  |  |  |  |  |
| 8. T3 Depressive symptoms | 8.42 | 6.31 | –.31** | .45** | –.49** | –.51** | –.61** | .71** | .79** |  |  |  |
|  |  |  | [–.38, –.25] | [.39, .50] | [–.55, –.43] | [–.58, –.44] | [–.65, –.56] | [.67, .74] | [.75, .82] |  |  |  |
|  |  |  |  |  |  |  |  |  |  |  |  |  |
| 9. T1 Anxiety Symptoms | 8.12 | 6.18 | –.26** | .60** | –.62** | –.51** | –.49** | .80** | .65** | .63** |  |  |
|  |  |  | [–.29, –.22] | [.57, .62] | [–.65, –.59] | [–.56, –.46] | [–.55, –.44] | [.79, .82] | [.61, .69] | [.59, .68] |  |  |
|  |  |  |  |  |  |  |  |  |  |  |  |  |
| 10. T2 Anxiety Symptoms | 6.72 | 5.82 | –.29** | .51** | –.52** | –.59** | –.51** | .65** | .83** | .70** | .74** |  |
|  |  |  | [–.35, –.22] | [.46, .56] | [–.57, –.47] | [–.63, –.54] | [–.58, –.44] | [.61, .69] | [.81, .85] | [.65, .74] | [.70, .77] |  |
|  |  |  |  |  |  |  |  |  |  |  |  |  |
| 11. T3 Anxiety Symptoms | 7.30 | 6.00 | –.33** | .49** | –.48** | –.49** | –.60** | .63** | .70** | .81** | .71** | .78** |
|  |  |  | [–.39, –.26] | [.44, .55] | [–.54, –.42] | [–.56, –.42] | [–.65, –.55] | [.58, .67] | [.65, .75] | [.79, .84] | [.67, .74] | [.74, .81] |
|  |  |  |  |  |  |  |  |  |  |  |  |  |

*Note.* T1 occurred between May 5, 2020 and September 30, 2020, T2 occurred between August 5, 2020 and January 29, 2021, and T3 occurred between November 5, 2020 and April 9, 2021. Intolerance of uncertainty was measured with the 12-item Intolerance of Uncertainty Scale – Short Form (Carleton et al., 2007). Psychological flexibility was measured with the 8-item Mental Flexibility Questionnaire – State (Parsons et al., 2022). (Parsons et al., 2022). Depression was measured with the 8-item Patient Health Questionnaire (Kroenke et al., 2009) and anxiety was measured with the 7-item Generalized Anxiety Disorder Scale (Spitzer et al., 2006).

**Measures**

***COVID-19 Risk***

The COVID-19 risk items were separated into groups A and B. Group A items were scored as 4 for death in close kin/relation (i.e., parent, child, sibling, grandparent, or friend) and 3 for hospitalization in self or close kin/relation. Group B items were scored as 2 for death in another kin/relation, 1.5 for hospitalization in another kin/relation, 1 for quarantine due to diagnosis in self, and 0.5 for diagnosis in any other person and quarantine for reasons other than diagnosis. To compute the COVID-19 risk score, the sum of the group A items was added to the highest-value group B item.

**Data Analysis**

General linear models were analyzed using the stats package (R Core Team, 2013), mixed-effects models were analysed using the lme4 package (Bates et al., 2015), and mediation models were analysed using the lavaan package (Rosseel, 2012). Data were visualized using the ggplot2 package (Wickham, 2016), interactions package (Long, 2021), and the raincloud plot function (Allen et al., 2021).

**Results**

**Hypothesis 1: Age-Related Differences in Intolerance of Uncertainty and Psychological Flexibility**

**SM Table 3**

*Age-Related Differences in Intolerance of Uncertainty and Psychological Flexibility at T1*

| Predictor |  | T1 intolerance of uncertainty | | |  |  | T1 psychological flexibility | | |  |
| --- | --- | --- | --- | --- | --- | --- | --- | --- | --- | --- |
|  | *b* | *SE* | 95% CI | *t*(2276) | *P* | *b* | *SE* | 95% CI | *t*(2127) | *p* |
| Intercept | 36.70 | 1.62 | 33.52, 39.88 | 22.66 | <.001 | 27.64 | 0.64 | 26.39, 28.89 | 43.33 | <.001 |
| Age group (linear) | **3.55** | **1.27** | **1.05, 6.05** | **2.79** | **.005** | **2.05** | **0.21** | **1.64, 2.47** | **9.70** | **<.001** |
| Age group (quadratic) | **–1.32** | **0.25** | **–1.80, –0.83** | **–5.34** | **<.001** |  |  |  |  |  |
| T1 COVID risk | **0.30** | **0.13** | **0.04, 0.56** | **2.28** | **.023** | –0.17 | 0.10 | –0.36, 0.03 | –1.63 | .103 |
|  | Observations 2280  R^2^ / R^2^ adjusted 0.065 / 0.064 | | | | | Observations 2126  R^2^ / R^2^ adjusted 0.044 / 0.043 | | | | |

*Note.* T1 occurred between May 5, 2020 and September 30, 2020. Intolerance of uncertainty was measured with the 12-item Intolerance of Uncertainty Scale – Short Form (Carleton et al., 2007). Psychological flexibility was measured with the 8-item Mental Flexibility Questionnaire – State (Parsons et al., 2022). The following age groups were used: early-to-mid adolescent (11–17 years), late adolescent (18–24 years), adult (25–64 years), and older adult (65+ years). CI = confidence interval. Bold indicates *p*-value < .05.

**Hypothesis 2: The Role of Intolerance of Uncertainty and Psychological Flexibility in the Association Between Age and Mental Health Problems**

**SM Table 4**

*The Relationship Between Age, Intolerance of Uncertainty, Psychological Flexibility, and Mental Health at T1*

| Predictor |  |  | T1 depression |  |  |  |  | T1 anxiety |  |  |
| --- | --- | --- | --- | --- | --- | --- | --- | --- | --- | --- |
|  | *b* | *SE* | 97.5% CI | *t* | *p* | *b* | *SE* | 97.5% CI | *t* | *p* |
| Intolerance of uncertainty model | | | | | | | | | | |
| Intercept | 2.43 | 1.57 | –1.08, 5.95 | 1.55 | .121 | –0.78 | 1.48 | –4.10, 2.54 | –0.52 | .601 |
| Age group | **–1.31** | **0.51** | **–2.46, –0.17** | **–2.57** | **.010** | **–1.08** | **0.48** | **–2.16, –0.01** | **–2.25** | **.024** |
| T1 intolerance of uncertainty | **0.33** | **0.04** | **0.24, 0.42** | **8.22** | **<.001** | **0.34** | **0.04** | **0.26, 0.43** | **8.98** | **<.001** |
| T1 COVID risk | **0.17** | **0.07** | **0.01, 0.32** | **2.44** | **.015** | **0.20** | **0.06** | **0.05, 0.34** | **3.03** | **.002** |
| Age group x T1 intolerance of uncertainty | –0.01 | 0.01 | –0.04, 0.02 | –0.92 | .358 | –0.00 | 0.01 | –0.03, 0.03 | –0.23 | .820 |
| Psychological flexibility model | | | | | | | | | | |
| Intercept | 26.90 | 1.77 | 22.93, 30.88 | 15.17 | <.001 | 22.94 | 1.72 | 19.09, 26.79 | 13.37 | <.001 |
| Age group | –0.72 | 0.61 | –2.09, 0.65 | –1.18 | .237 | 0.40 | 0.59 | –0.93, 1.72 | 0.67 | .503 |
| T1 psychological flexibility | **–0.38** | **0.05** | **–0.50, –0.26** | **–7.02** | **<.001** | **–0.34** | **0.05** | **–0.46, –0.22** | **–6.48** | **<.001** |
| T1 COVID risk | **0.18** | **0.07** | **0.03, 0.33** | **2.63** | **.009** | **0.18** | **0.07** | **0.03, 0.33** | **2.77** | **.006** |
| Age group x T1 psychological flexibility | –0.03 | 0.02 | –0.07, 0.01 | –1.69 | 0.092 | **–0.05** | **0.02** | **–0.09, –0.01** | **–2.82** | **0.005** |

*Note.* T1 occurred between May 5, 2020 and September 30, 2020. Intolerance of uncertainty was measured with the 12-item Intolerance of Uncertainty Scale – Short Form (Carleton et al., 2007). Psychological flexibility was measured with the 8-item Mental Flexibility Questionnaire– State (Parsons et al., 2022). Depression was measured with the 8-item Patient Health Questionnaire (Kroenke et al., 2009) and anxiety was measured with the 7-item Generalized Anxiety Disorder Scale (Spitzer et al., 2006). The following age groups were used: early- to-mid adolescent (11–17 years), late adolescent (18–24 years), adult (25–64 years), and older adult (65+ years). CI = confidence interval. Bold indicates p-value < .025. Intolerance of uncertainty on depression model: *F*(4, 2274) = 282.5, *p* < .001, *R^2^* = .33; psychological flexibility on depression model: *F*(4, 2120) = 359.7, *p* < .001, *R*^2^ = .40. Intolerance of uncertainty on anxiety model: *F*(4, 2273) = 345.1, *p* < .001, *R^2^* = .38; psychological flexibility on anxiety model: *F*(4, 2119) = 370.4, *p* < .001, *R*^2^ = .41.

**SM Table 5**

*The Relationship Between Age, Intolerance of Uncertainty, Psychological Flexibility, and Mental Health Across Time*

| Predictor |  |  | Depression |  |  |  |  | Anxiety |  |  |
| --- | --- | --- | --- | --- | --- | --- | --- | --- | --- | --- |
|  | *b* | *SE* | 97.5% CI | *t* | *p* | *b* | *SE* | 97.5% CI | *t* | *p* |
| Intolerance of uncertainty model | | | | | | | | | | |
| Intercept | 2.71 | 1.52 | –0.68, 6.11 | 1.79 | .073 | –0.25 | 1.41 | –3.42, 2.92 | –0.18 | .857 |
| Time | **–0.36** | **0.08** | **–0.53, –0.19** | **–4.70** | **<.001** | **–0.31** | **0.07** | **–0.48, –0.15** | **–4.30** | **<.001** |
| T1 intolerance of uncertainty | **0.34** | **0.04** | **0.25, 0.42** | **8.59** | **<.001** | **0.34** | **0.04** | **0.26, 0.42** | **9.37** | **<.001** |
| Age group | **–1.15** | **0.49** | **–2.25, –0.05** | **–2.35** | **.019** | –0.97 | 0.46 | –2.00, 0.05 | –2.13 | .033 |
| COVID risk | **0.13** | **0.05** | **0.03, 0.24** | **2.78** | **.005** | **0.15** | **0.05** | **0.05, 0.26** | **3.44** | **.001** |
| Age group x T1 intolerance of uncertainty | –0.02 | 0.01 | –0.05, 0.01 | –1.36 | .174 | –0.01 | 0.01 | –0.04, 0.02 | –0.66 | .508 |
| Psychological flexibility model | | | | | | | | | | |
| Intercept | 26.26 | 1.43 | 23.04, 29.47 | 18.30 | <.001 | 22.13 | 1.38 | 19.03, 25.23 | 16.03 | <.001 |
| Time | **–0.42** | **0.08** | **–0.60, –0.25** | **–5.58** | **<.001** | **–0.38** | **0.07** | **–0.55, –0.22** | **–5.21** | **<.001** |
| Psychological flexibility | **–0.34** | **0.04** | **–0.44, –0.24** | **–7.83** | **<.001** | **–0.29** | **0.04** | **–0.38, –0.20** | **–6.93** | **<.001** |
| Age group | **–1.18** | **0.49** | **–2.28, –0.08** | **–2.41** | **.016** | –0.24 | 0.47 | –1.30, 0.81 | –0.52 | .605 |
| COVID risk | **0.16** | **0.05** | **0.05, 0.26** | **3.35** | **.001** | **0.17** | **0.04** | **0.07, 0.27** | **3.70** | **<.001** |
| Age group x psychological flexibility | –0.02 | 0.01 | –0.05, 0.01 | –1.35 | .176 | **–0.04** | **0.01** | **–0.07, –0.00** | **–2.54** | **.011** |

*Note.* T1 occurred between May 5, 2020 and September 30, 2020, T2 occurred between August 5, 2020 and January 29, 2021, and T3 occurred between November 5, 2020 and April 9, 2021. Intolerance of uncertainty was measured with the 12-item Intolerance of Uncertainty Scale – Short Form (Carleton et al., 2007). Psychological flexibility was measured with the 8-item Mental Flexibility Questionnaire – State (Parsons et al., 2022). Depression was measured with the 8-item Patient Health Questionnaire (Kroenke et al., 2009) and anxiety was measured with the 7-item Generalized Anxiety Disorder Scale (Spitzer et al., 2006). The following age groups were used: early-to-mid adolescent (11–17 years), late adolescent (18–24 years), adult (25–64 years), and older adult (65+ years). CI = confidence interval. Bold indicates *p*-value < .025. Intolerance of uncertainty on depression model: conditional *R^2^* = .75, marginal *R^2^* = .31; psychological flexibility on depression model: conditional *R^2^* = .73, marginal *R^2^* = .34. Intolerance of uncertainty on anxiety model: conditional *R^2^* = .75, marginal *R^2^* = .35; psychological flexibility on anxiety model: conditional *R^2^* = .72, marginal *R^2^* = .34.
